# Modified tailoring the electronic phase and emergence of midstates in impurity-imbrued armchair graphene nanoribbons

**DOI:** 10.1038/s41598-019-47015-9

**Published:** 2019-07-23

**Authors:** Nguyen D. Hien, Kavoos Mirabbaszadeh, Masoumeh Davoudiniya, Bui D. Hoi, Le T. T. Phuong, Mohsen Yarmohammadi

**Affiliations:** 1 Laboratory of Magnetism and Magnetic Materials, Advanced Institute of Materials Science, Ton Duc Thang University, Ho Chi Minh City, Vietnam; 2 Faculty of Applied Sciences, Ton Duc Thang University, Ho Chi Minh City, Vietnam; 3 Department of Energy Engineering and Physics, Amirkabir University of Technology, Tehran, Iran; 4 Center for Theoretical and Computational Physics and Department of Physics, University of Education, Hue University, Hue City, Vietnam

**Keywords:** Electronic properties and devices, Condensed-matter physics

## Abstract

We theoretically address the electronic structure of mono- and simple bi-layer armchair graphene nanoribbons (AGNRs) when they are infected by extrinsic charged dilute impurity. This is done with the aid of the *modified* tight-binding method considering the *edge effects* and the Green’s function approach. Also, the interplay of host and guest electrons are studied within the full self-consistent Born approximation. Given that the main basic electronic features can be captured from the electronic density of states (DOS), we focus on the perturbed DOS of lattices corresponding to the different widths. The modified model says that there is no metallic phase due to the edge states. We found that the impurity effects lead to the emergence of midgap states in DOS of both systems so that a semiconductor-to-semimetal phase transition occurs at strong enough impurity concentrations and/or impurity scattering potentials. The intensity of semiconductor-to-semimetal phase transition in monolayer (bilayer) ultra-narrow (realistic) ribbons is sharper than bilayers (monolayers). In both lattices, electron-hole symmetry breaks down as a result of induced-impurity states. The findings of this research would provide a base for future experimental studies and improve the applications of AGNRs in logic semiconductor devices in industry.

## Introduction

Discovering graphene as the first two-dimensional material in 2004 by isolating crystal graphite sheets^1^ opened a new window in nanotechnology science. The energy dispersion relation of carriers in graphene behaves linearly at low energies and the corresponding DOS illustrates a symmetric V-shape diagram at low temperatures as well. Due to the high thermal conductivity^2^ and electronic mobility^3^ of Dirac fermions in graphene, the graphene-like nanostructures such as graphene nanoribbon (GNR)^4^ have been widely used in micro- and nano-electronic devices^5,6^. However, the application of graphene is limited in logic electronics because of the nature of zero band gap in pristine graphene^7^; thus, it is essential to find a way by which not only the band gap can be adjusted but also the mobility of carriers would not be intensely changed. Geometrically, GNRs cut from a hexagonal lattice of graphene in two edged shapes, including armchair GNR (AGNR) and zigzag GNR (ZGNR). The electronic properties of graphene around the Fermi-level are strongly influenced by the chemical modification or the edge structure^8^. For instance, the ZGNRs only show the metallic behaviors whereas the metallic or semiconducting phase of AGNRs depend critically on the ribbon width^9^. Also, as stated in ref.^10^, the ZGNRs and AGNRs behave differently in the presence of an electric field. The results of ref.^10^ showed that when applying an electric field for the metallic ZGNRs the band gap opens whilst the band gap of AGNRs reduces.

In addition to monolayer GNRs, the bilayer GNRs have also drawn attention in different areas such as theoretical^11,12^ and experimental^13^ research as well as several nano-electromechanical devices^14,15^. Also, as it has been reported, an effective method to tune the band gap of GNRs is to stack two monolayer GNRs to form a bilayer GNR^16^. Despite small band gap of bilayer GNRs in comparison with monolayer GNRs^17^, these are widely used in nanoelectronic devices due to their band gap adjustability in the presence of perpendicular electron field^17^ and the low sensitivity to low frequencies^18^. Moreover, we know that the stacking configuration influences the electronic properties of bilayer GNRs^19^. Generally, the bilayer GNRs have two main stacking configurations, including AA– and AB–stacking configurations, of which, the AB-stacked is energetically the most stable configuration. However, contrary to the case of AB–stacked bilayer GNRs, there are few works on AA–stacked bilayer GNRs.

Several groups deal with the effects of defects on the transport properties of mono- and bi-layer graphene^10,20–22^. Demin Yin, *et al*.^23^ have reported that the effective quantum transport channels in mono-bi-mono-layer graphene junctions are more than the full monolayer GNRs. Chang *et al*.^24^ experimentally observed that doping hexagonal boron nitride in the graphene film leads to the appearance of a remarkable band gap equal to 600 meV in the graphene. Similarly, A. Lherbier *et al*.^25^ theoretically indicated that the band gap of graphene sheets is tuned by doping nitrogen due to the breaking of the graphene sublattice symmetry. In addition, in ref.^26^ the authors theoretically found out that the electronic phase transition of monolayer armchair graphene-like nanoribbons can be adjusted by applying extrinsic impurities.

In the present paper, we analytically study the impacts of charged dilute impurity on electronic properties of armchair monolayer and AA–stacked bilayer AGNRs. Since the DOS around the Fermi energy can provide basic information on the transport properties, we investigate DOS of these lattices in the absence and presence of charged dilute impurity. The impurity is doped randomly and distributes on both two sublattices equally. However, the results only for randomly doping on one of the sublattices is investigated as well. We use the modified tight-binding Hamiltonian, the Born approximation, and the Green’s function theory to achieve the expected considerable findings. There are many works where the electronic properties of AGNRs have been studied in the presence if different types of adsorbates. However, most of them have not considered randomly impurity doped case, which is a more general case in experiment.

The rest of the paper is structured as follows: Sec. 2 gives a brief overview of our model, the non-interacting Green’s function, and unperturbed DOS. Sec. 3 presents interacting Green’s function and perturbed DOS aimed at investigating the effects of impurity on electronic properties of monolayer and AA–stacked bilayer AGNRs. In Sec. 4 we assess the numerical results and phase tailoring in mono- and bi-layer GNRs. Eventually, our conclusions are included in Sec. 5.

## Model and Unperturbed DOS

**Figure 1:**
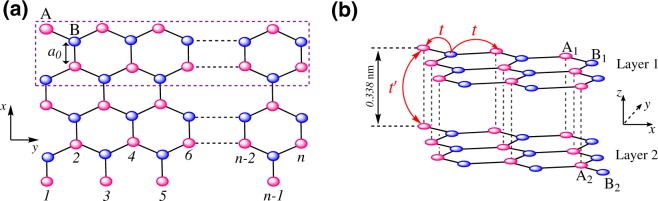
Sketch of the (**a**) top view of MLAGNR and the (**b**) side view of BLAGNR with translational symmetry along *y*–direction. The dashed rectangle in (**a**) delimits the unit cell. The intralayer and interlayer hopping parameters are labeled by *t* and *t*′ in (**b**).

In this section, we intend to describe the carrier dynamics for both monolayer and simple bilayer AGNRs. In Fig. 1(a), we consider a pristine monolayer AGNR (MLAGNR) of width $\sqrt{3}n{a}_{0}$ (${a}_{0}\simeq 1.42$ Å being the interatomic distance between carbon atoms) wherein the rectangle delimits the unit cell. Also, the sketch of simple bilayer AGNR (BLAGNR) is illustrated in panel (b). We describe the electrons in both systems using the tight-binding (TB) Hamiltonian model, respectively, as^27–30^

1a $${\hat{ {\mathcal H} }}_{{\rm{MLAGNR}}}^{{\rm{TB}}}=-\,t\sum _{\langle i,j\rangle }[{\hat{a}}_{i}^{\dagger }{\hat{b}}_{j}+{\rm{H}}{\rm{.c}}{\rm{.}}],$$

1b $${\hat{ {\mathcal H} }}_{{\rm{BLAGNR}}}^{{\rm{TB}}}=-\,\sum _{\langle i,j\rangle }\sum _{l,l^{\prime} }[t{\hat{a}}_{li}^{\dagger }{\hat{b}}_{lj}+t^{\prime} {\hat{a}}_{li}^{\dagger }{\hat{a}}_{l^{\prime} j}+{\rm{H}}{\rm{.c}}\mathrm{.]}\mathrm{.}$$

which are on the basis of envelope wave functions *ψ*_A_(*k*_*x*_, *k*_*y*_) and *ψ*_B_(*k*_*x*_, *k*_*y*_) for sublattices A and B, respectively. On the other hand, ${\hat{a}}_{i}^{\dagger }$ and ${\hat{b}}_{j}$ are electron creation and annihilation operators at atomic site *i* and *j* of sublattice A and B, respectively. The negative sign of *t* and *t*′ corresponding to the *intralayer* and *interlayer* hoppings, respectively, originate from the proper bonding of *p*_*z*_–orbitals in graphene^31^. The value of these hopping parameters in our calculations are taken from ref.^28^, $t\simeq 3$ eV and $t^{\prime} \simeq 0.4$ eV. Also, *l* and *l*′ are indexed for layer, and the term H.c. in both Hamiltonians stands for the Hermitian conjugate of operators.

To achieve the dispersion energy relations for both lattices, we use a Fourier transformation along the translationally invariant *x* axis. Before entering into the transformation, we simplify the problem. We assume that each unit cell can be characterized with an index *m* and sublattice A_*p*_/B_*p*_ [*p* ∈ (1, n)]. Thereby, we use the expression

2 $${\hat{c}}^{\dagger }({k}_{x},{k}_{y})=\frac{1}{\sqrt{M}}\sum _{m\mathrm{=1}}^{M}\sum _{p\mathrm{=1}}^{n}{e}^{{\mathtt{i}}{k}_{x}{x}_{m}}{\psi }_{c}(p,{k}_{y}){\hat{c}}^{\dagger }(p,m),$$

for $\hat{c}=\hat{a}$ or $\hat{b}$. In this expression, *M* is the number of unit cells, *x*_*m*_ is the position of site *m*, and *k*_*x*_ is the momentum along the *x* axis. It should be noted that the periodic boundary conditions are applied along the *y* axis. In Eq. (2), one can consider ${\psi }_{{\rm{A}}/{\rm{B}}}(p,{k}_{y})=\,\sin (\sqrt{3}{k}_{y}{a}_{0}p/2)$ with the discretized wave-vector ${k}_{y}=2z\pi /(\sqrt{3}{a}_{0}[n+1])$^32^. Substituting the Hamiltonians in terms of the Fourier transformed operators described in Eq. (2) into the Schrödinger equation gives the eigenvalues [dispersion energy relations] for MLAGNRs and BLAGNRs, respectively, given by

3a $${ {\mathcal E} }_{\nu }^{{\rm{Mono}}}({k}_{x},z)=\nu |\varphi ({k}_{x},z)|,$$

3b $${ {\mathcal E} }_{\nu ,\sigma }^{{\rm{Bi}}}({k}_{x},z)=\nu \sqrt{2\varphi ({k}_{x},z){\varphi }^{\ast }({k}_{x},z)+{t}^{^{\prime} 2}+\sigma \sqrt{{t}^{^{\prime} 4}+4{t}^{2}{t}^{^{\prime} 2}}\varphi ({k}_{x},z){\varphi }^{\ast }({k}_{x},z)}\mathrm{.}$$

where $\varphi ({k}_{x},z)=-\,t\mathrm{[2}\exp ({\mathtt{i}}{k}_{x}{a}_{0}/\mathrm{2)}\,\cos (\sqrt{3}{k}_{y}{a}_{0}/2)+\exp (\,-\,{\mathtt{i}}{k}_{x}{a}_{0})]$ is the momentum-dependent structure factor. $\nu =\pm$ stands for valence (−) and conduction (+) band while $\sigma =\pm$ is for upper (+) and lower (−) layer in bilayer case.

It has been shown that the *n*-AGNRs are semiconductors with energy gaps which decrease as a function of increasing ribbon widths of which the variations in energy gap, however, exhibit three distinct family behaviors including *n* = 3*p*, *n* = 3*p* + 1 and *n* = 3*p* + 2 (where *p* is a positive integer). The energy gaps obtained by the simple TB model described above are quite different from those by first-principles calculations^33^. Our tight-binding model above shows that using a constant nearest neighbor hopping integral $t\simeq 3$ eV, *n*-AGNRs is metallic if *n* = 3*p* + 2 or otherwise, it is semiconducting, in agreement with previous works^34–39^. However, for the first-principles calculations, there are no metallic nanoribbons. A determining factor in the semiconducting behavior of *n*-AGNR is quantum confinement and the edge effects which force the (3*p* + 2)-AGNRs (predicted to be metallic by TB model above) to be semiconductors. To see the consequence of such effects more clearly, we have introduced a lattice model which is equivalent to the AGNRs within the TB approximation^34–37^. The new Hamiltonian of the model is given by

4a $${\hat{ {\mathcal H} }}_{{\rm{MLAGNR}}}^{{\rm{LDA}}}=-\,\sum _{\langle i,j\rangle }{t}_{i}^{\perp }{\hat{a}}_{i}^{\dagger }{\hat{b}}_{j}-\sum _{i}{t}_{i,i+1}^{\parallel }{\hat{a}}_{i}^{\dagger }{\hat{b}}_{i+1}+{\rm{H}}{\rm{.c}}{\rm{.}},$$

4b $${\hat{ {\mathcal H} }}_{{\rm{BLAGNR}}}^{{\rm{LDA}}}=-\,\sum _{\langle i,j\rangle ,l}{t}_{i}^{\perp }{\hat{a}}_{li}^{\dagger }{\hat{b}}_{lj}-\sum _{i,l}{t}_{i,i+1}^{\parallel }{\hat{a}}_{il}^{\dagger }{\hat{b}}_{i+\mathrm{1,}l}+t^{\prime} \sum _{l,l^{\prime} }{\hat{a}}_{li}^{\dagger }{\hat{a}}_{l^{\prime} j}+{\rm{H}}{\rm{.c}}\mathrm{..}$$

where ${t}_{i}^{\perp }$ and ${t}_{i,i+1}^{\parallel }$ denote the nearest neighbor hopping integrals within each leg and between the legs, respectively. Hence, considering the simplest but essential variation from the exact solvable model to approximate the realistic situations with first-principles, we assume that ${t}_{1}^{\perp }={t}_{n}^{\perp }=\mathrm{(1}+\delta )t$, ${t}_{i\in \mathrm{\{2,...,}n-\mathrm{2\}}}^{\perp }=t$ and ${t}_{i,i+1}^{\parallel }=t$ where with $\delta \simeq 0.19$, the calculated gaps obtained using the new Hamiltonian model are in good agreement with the local density approximation results in ref.^33^. The resulting energy gaps to the first order in *δ* are given by

5a $${ {\mathcal E} }_{g}^{n=3k}\simeq |{ {\mathcal E} }_{+,\sigma }^{\mathrm{Mono}/\mathrm{Bi}}{|}_{n=3k}-{ {\mathcal E} }_{-,\sigma }^{\mathrm{Mono}/\mathrm{Bi}}{|}_{n=3k}|-\frac{8\delta t}{3k+1}{\sin }^{2}(\frac{k\pi }{3k+1}),$$

5b $${ {\mathcal E} }_{g}^{n\mathrm{=3}k+1}\simeq |{ {\mathcal E} }_{+,\sigma }^{\mathrm{Mono}/\mathrm{Bi}}{|}_{n=3k+1}-{ {\mathcal E} }_{-,\sigma }^{\mathrm{Mono}/\mathrm{Bi}}{|}_{n\mathrm{=3}k+1}|+\frac{8\delta t}{3k+2}{\sin }^{2}(\frac{(k+\mathrm{1)}\pi }{3k+2}),$$

5c $${ {\mathcal E} }_{g}^{n=3k+2}\simeq |{ {\mathcal E} }_{+,\sigma }^{\mathrm{Mono}/\mathrm{Bi}}{|}_{n=3k+2}-{ {\mathcal E} }_{-,\sigma }^{\mathrm{Mono}/\mathrm{Bi}}{|}_{n=3k+2}|+\frac{2\delta t}{k+1}\mathrm{.}$$

This implies that the 19% increase of the hopping integrals between carbon atoms at the edges opens the gaps of the (3*p* + 2)-AGNRs and decreases (increases) the gaps of 3*p*-AGNRs [(3*p* + 1)-AGNRs]. This happens for the case of *δ* = 0.12 in ref.^33^.

**Figure 2:**
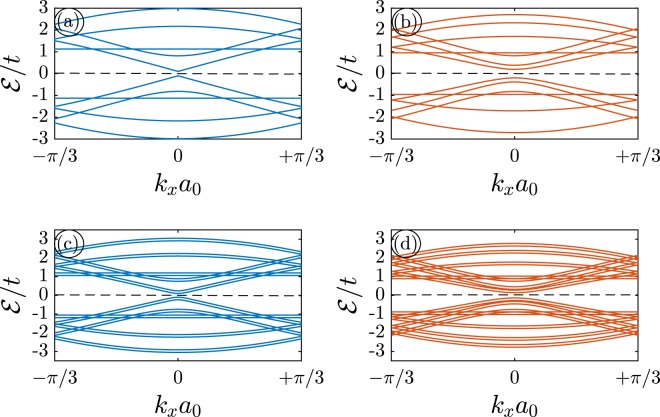
The electronic band structure of clean AGNRs with different widths for (**a**) 5–MLAGNR, (**b**) 7–MLAGNR, (**c**) 5–BLAGNR, and (**d**) 7–BLAGNR. We set the Fermi level to zero (black horizontal dashed line at ${\mathcal E} /t=0$). All panels show the semiconducting phase for both mono- and bi-layer AGNRs.

The electronic band structure of two different widths of MLAGNRs and BLAGNRs are presented in Fig. 2 in order to show the band gap-dependent phase of the systems. For simplicity and to have non-messy bands in panels, *n* = 5 and *n* = 7 are chosen arbitrarily without any physical reason behind them. Considering the edge effects there is no band touching in electronic band structures and eventually no degenerate states in the electronic DOS, as will be shown later in DOS curves. This implies that there are no metallic nanoribbons and all are semiconductors. Although focusing on the dispersion energy band behaviors is one of the ways to study the electronic properties of materials, in this work, we are focused on the electronic DOS quantity.

To derive the electronic DOS we need an effective tool to describe the electronic correlations between carriers of different sublattices. To this end, we use the Green’s function approach. Moreover, with the aid of the Matsubara formalism^40^ the non-interacting Green’s function elements are given by

6 $${G}_{\alpha \beta }^{0}({k}_{x},\tau )=-\langle {{\mathbb{T}}}_{\tau }[{\hat{c}}_{\alpha }({k}_{x},\tau ){\hat{c}}_{\beta }^{\dagger }({k}_{x}\mathrm{,0)}]\rangle ,$$

where *α* and *β* refer to each sublattice A and B. The symbol ${\mathbb{T}}$ and *τ* stand for the time ordering operator and the imaginary time, respectively. However, we need the Fourier transformation of these elements in the momentum-energy space which can be obtained with the following relation,

7 $${G}_{\alpha \beta }^{0}({k}_{x}, {\mathcal E} +{\mathtt{i}}{0}^{+})={\int }_{0}^{\mathrm{1/}{k}_{{\rm{B}}}T}{e}^{[ {\mathcal E} +{\mathtt{i}}{0}^{+}]\tau }{G}_{\alpha \beta }^{0}({k}_{x},\tau )d\tau ,$$

where ${\mathcal E} +{\mathtt{i}}{0}^{+}={\mathtt{i}}{\omega }_{k}=(2k+1){k}_{{\rm{B}}}T$ (*k* is a positive integer number) is the Fermionic Matsubara frequency with 0^+^ = 10 meV in numerical computations, *k*_B_ is the Boltzmann constant, and *T* is the temperature. The Green’s function elements help us to calculate the electronic DOS using the trace over the imaginary diagonal elements, i.e. ${G}_{\alpha \alpha }^{0}({k}_{x}, {\mathcal E} +{\mathtt{i}}{0}^{+})$:

8 $${{\mathscr{D}}}^{0}( {\mathcal E} )=-\,\frac{1}{\pi M}\sum _{\alpha }\sum _{{k}_{x}\in {\rm{FBZ}}}{\rm{Im}}[{G}_{\alpha \alpha }^{0}({k}_{x}, {\mathcal E} +i{0}^{+})],$$

So far, we have focused on the unperturbed lattices. However, our main aim in the present paper is exploring the effect of charged impurity doping on the electronic properties of mono- and bi-layer AGNRs using the electronic DOS. In what follows, we will focus on this by studying the interaction between the host and guest electrons.

## Dilute Charged Impurity Effects

As stated in the introduction, the main target of the current study is to investigate the impacts of doping *randomly dilute* charged impurity on the electronic properties of MLAGNR or BLAGNR. In our formalism, we generally address short-range impurity because the Coulomb impurity behaves as short-range in AGNRs due to screening. Also, the impurity is modelled as a *δ* function potential and by considering *u* as a constant in momentum space, the below expression can be defined as the impurity potentials for MLAGNR and BLAGNR, respectively

9 $${\hat{U}}^{{\rm{MLAGNR}}}=u(\begin{array}{cc}1 & 0\\ 0 & 0\end{array}),\,{\hat{U}}^{{\rm{BLAGNR}}}=u(\begin{array}{cccc}1 & 0 & 0 & 0\\ 0 & 0 & 0 & 0\\ 0 & 0 & 1 & 0\\ 0 & 0 & 0 & 0\end{array}),$$

Non-zero elements in above matrix denote the place of the impurity. Here, there are one and two non-zero elements where the element in first row and first column in both $\hat{U}$ shows that the impurity resides on the *A*/*B* atom in MLAGNR. On the other hand, the element in third row and third column of ${\hat{U}}^{{\rm{BLAGNR}}}$ denotes that *A*/*B* atom of second layer in BLAGNR is in vicinity of the charged impurity. Furthermore, *u* → ∞ implies the vacancies. In order to study the electronic properties of *dilute* charge impurity induced-MLAGNR or -BLAGNR, we assume that the charged impurities are *randomly* doped on *A* and *B* sublattices equally or on only *A*/*B* sublattice. Eventually, the final conclusion can be obtained by calculating the average over all configurations of charged impurities in the system. It is fundamental to note that in the calculations of short range impurity and small value for *u*, the Born approximation is mostly used. Whilst in the case of forming bound states^40^ and *dilute* impurity and vacancies^41^, T-matrix approximation is applied.

Thus, using the Matsubara frequency^42^, the Born approximation in the scattering theory and T-matrix^42^, the full Green’s function in momentum space can be extracted via

10 $$\hat{G}({{\bf{k}}}_{1},{{\bf{k}}}_{2}, {\mathcal E} )={\hat{G}}^{\mathrm{(0)}}({{\bf{k}}}_{1}-{{\bf{k}}}_{2}, {\mathcal E} )+{\hat{G}}^{\mathrm{(0)}}({{\bf{k}}}_{1}, {\mathcal E} ){T}_{imp}({{\bf{k}}}_{1},{{\bf{k}}}_{2}, {\mathcal E} ){\hat{G}}^{\mathrm{(0)}}({{\bf{k}}}_{2}, {\mathcal E} ),$$

in which the ${\hat{T}}_{imp}$ matrix is satisfied by the self-consistent relation

11 $${\hat{T}}_{imp}({{\bf{k}}}_{1},{{\bf{k}}}_{2}, {\mathcal E} )=\hat{U}({{\bf{k}}}_{1},{{\bf{k}}}_{2})+\sum _{{\bf{k}}^{\prime} }\,\hat{U}({{\bf{k}}}_{1},{\bf{k}}^{\prime} ){\hat{G}}^{\mathrm{(0)}}({\bf{k}}^{\prime} , {\mathcal E} ){\hat{T}}_{imp}({\bf{k}}^{\prime} ,{{\bf{k}}}_{2}, {\mathcal E} ),$$

leading to

12 $${\hat{T}}_{imp}( {\mathcal E} )=\frac{{\nu }_{i}}{\hat{I}-\frac{{\nu }_{i}}{{N}_{i}}\sum _{{\bf{k}}\in FBZ}\,{\hat{G}}^{\mathrm{(0)}}({\bf{k}}, {\mathcal E} )},$$

where *v*_i_ (*N*_*i*_) is the scattering potential (number) of the impurity. Further, the impurity induced-DOS elements are introduced by the following expression

13 $$\delta {\mathscr{D}}({\bf{p}}, {\mathcal E} )=\frac{-1}{{N}_{a}\pi }\sum _{{\bf{k}}}\,[\delta \hat{G}({\bf{k}},{\bf{k}}+{\bf{p}})-\delta {\hat{G}}^{\mathrm{(0)}}({\bf{k}},{\bf{k}}+{\bf{p}})],$$

in which

14 $$\delta \hat{G}({\bf{k}},{\bf{k}}+{\bf{p}})=\hat{G}({\bf{k}},{\bf{k}}+{\bf{p}})-{\hat{G}}^{\mathrm{(0)}}({\bf{k}},{\bf{k}}+{\bf{p}}),$$

Finally, the electronic self-energy matrix is written as

15 $${\hat{T}}_{imp}({\bf{p}}, {\mathcal E} )=\hat{U}{n}_{i}{[1-\frac{\hat{U}}{{N}_{i}}\sum _{{\bf{k}}\in FBZ}{\hat{G}}_{\rho \rho }^{\mathrm{(0)}}({\bf{k}},{\bf{p}})]}^{-1}\mathrm{.}$$

where *n*_*i*_ refers to the impurity concentration. Also, the wave-vector induced by impurities to the host electrons is illustrated by **p**. Consequently, using the Dyson equation, the *perturbed* Green’s function is given by^42^

16 $$\hat{G}({\bf{k}}, {\mathcal E} )={\hat{G}}^{\mathrm{(0)}}({\bf{k}}, {\mathcal E} ){[1-{\hat{G}}^{\mathrm{(0)}}({\bf{k}}, {\mathcal E} ){\hat{T}}_{imp}({\bf{p}}, {\mathcal E} )]}^{-1}\mathrm{.}$$

Therefore, by computing the *disordered* DOS using *perturbed* Green’s function,

17 $${\mathscr{D}}( {\mathcal E} )=-\,\frac{1}{\pi M}\sum _{\alpha }\,\sum _{{k}_{x}\in {\rm{FBZ}}}\,\mathrm{Im}[{G}_{\alpha \alpha }({k}_{x}, {\mathcal E} +{\mathtt{i}}{0}^{+})],$$

we asses the impacts of impurity on electronic phase of AGNRs.

Here, we clarify what the reason of ignoring the coupling between impurity and carbon atom is in our paper. We know that the *π*-orbitals can participate in covalent bonding with adsorbates and the interaction between electrons in the *π* band of GNRs and the additional adsorbed atoms can be described using a tight-binding Hamiltonian^43^

18 $${\hat{ {\mathcal H} }}_{{\rm{e}}-{\rm{impurity}}}={\varepsilon }_{i}\sum _{o}\,{\hat{d}}_{o}^{\dagger }{\hat{d}}_{o}+{\gamma }_{i}[{\hat{c}}_{{\alpha }_{o}}^{\dagger }{\hat{d}}_{o}+{\rm{H}}{\rm{.c}}{\rm{.}}],$$

where ${\hat{d}}_{o}$ is the annihilation operator on the adsorbate site and ${\alpha }_{o}$ is the host position on the honeycomb lattice of AGNRs. The adsorbate density is parameterized by *n*_*i*_/*A*_*c*_, where *A*_*c*_ is the area per carbon atom in AGNRs.

However, the model Hamiltonian above can be justified by *first principle* calculations and the energies *ε*_*i*_ and *γ*_*i*_ depend strongly on the kind of the adsorbates chosen. These energies and also other energy scales (such as shifts of the graphene on-site energies and next-to-nearest neighbor couplings) obtain from first principle calculations and differ for different adsorbates^43–47^. Of course, it is possible to do this but we need to do a first principle calculation and consider different types of adsorbates in order to find *ε*_*i*_ and *γ*_*i*_, which is out of the scope of the present paper. Also, the impurity-carbon hopping integral could be scaled by carbon-carbon hopping integral *t*, but according to Eq. (18), the extra potential *γ*_*i*_ referring to the carbon-impurity interaction strength just shifts the total hopping integral energy to the lower and higher values depending on the sign of *γ*_*i*_/*t*. From the view of DFT, yes, this new interaction could cause buckling and orbital-hybridization that greatly modulates electronic properties in pristine structures, but in our theoretical formulation, this is just a energy shift and since all phases of nanoribbons after considering the edge effects are semiconductor, then it affects the band gap sizes only here.

Another remark can be refereed to the case of graphene with hydrogen adatoms in dense and dilute limits^48^. In addition to the interaction between the carbon atoms and impurities, first-principles calculations of the spin-orbit coupling in hydrogenated graphene shown that the chemisorbed hydrogen induces a giant local enhancement of spin-orbit coupling due to *sp*^3^ hybridization which depends strongly on the local lattice distortion. In the work mentioned, realistic minimal Hamiltonians are proposed that reproduce the relevant spin-orbit effects for both single-side semihydrogenated graphene and for a single hydrogen adatom in a large supercell. Note that this is not the case if only the hydrogenation is considered, implying that we have ignored the spin-orbit coupling as well in our formulation. It is worth mentioning that hydrogenation in graphene and other 2D materials lead to different physical features, for instance, a work by Zhang and Yan^49^ show that the weak overlapping between 3*pz* orbitals of neighbor silicon atoms leads to a very reactive surface, resulting in a more energetically stable semiconducting surface upon being fully hydrogenated. Half-hydrogenation breaks the extended *π*-bonding network of silicene, leaving the electrons in the unsaturated silicon atoms localized and unpaired, and thus it exhibits ferromagnetic semiconducting behavior with a band gap of 0.95 eV.

## Results and Discussions

This section is dedicated to analyze the electronic DOS of both MLAGNR and BLAGNR when the interaction between the host electrons and the guest ones (stemming from impurities) is considered. It is well-known that DOS provides main features of electronic phase of a material around the Fermi energy (taken as the zero energy ${\mathcal E} =0$), i.e. in the low-energy range. Generically, the electronic phases of materials considered in our work are divided into four category phases: (i) insulating, (ii) semiconducting, (iii) semimetallic, and (iv) metallic. Thereby, the insulator/semiconductor and metal/semimetal material phases are characterized by the zero and non-zero value of DOS at ${\mathcal E} =0$. In the present paper, we restrict ourselves to this for the phase desegregation. In our calculations, the Fermi energy is fixed at zero and does not shift with impurity.

In all plots follow, we plot the electronic DOS (in arbitrary units) versus normalized energy ${\mathcal E} /t$ in the range from −4 eV to +4 eV and −0.5 eV to +0.5 eV. We start with the *un-doped* systems to have an idea about their DOS shape. Then, we will deal with the effect of impurity under different conditions. It should be pointed out that the present work is based on three impurity doping ways: doping with (i) *the same* impurity atoms (different *n*_*i*_ and a fixed *v*_*i*_/*t*), (ii) *different* impurity atoms (different *v*_*i*_/*t* and a fixed *n*_*i*_) and (iii) the case when both *n*_*i*_ and *v*_*i*_/*t* are irrelevant. *n*_*i*_ = *x*% implies that the *x* percent of the whole unit cells are doped with the same impurity atoms. For this reason, *n*_*i*_ = 20% in our formulation for 1000 × 1000 simulated unit cell is dilute. This information makes the analysis of plots easier. One more thing should be clarified before entering into the analysis, which is the type of impurities. In our calculations, it is supposed to have donor charge impurity atoms, which implies that the results would happen in another way for the acceptor ones. For example, the *p*-doped semiconducting behavior will be changed to the *n*-doped one when switching the impurity from donor to acceptor.

**Figure 3:**
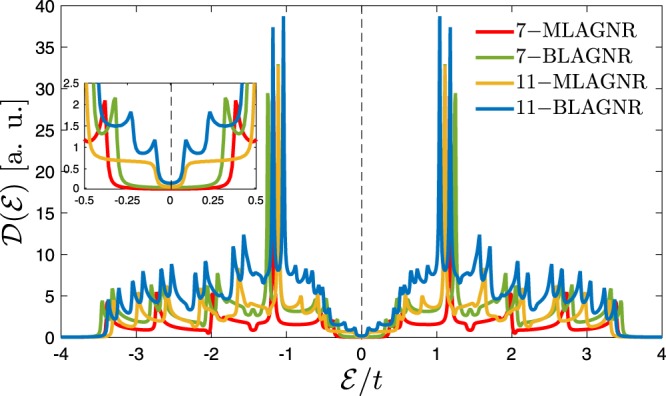
The electronic DOS of MLAGNR and BLAGNR. The systems are in the absence of impurity in this figure. The Fermi level is set to zero (black vertical dashed line at ${\mathcal E} /t=0$).

Let us start with Fig. 3, which presents DOS of clean, i.e. un-doped MLAGNR and BLAGNR for two arbitrary values of ribbon width, namely *n* = 7 and *n* = 11. As highlighted in the figure, for all cases the systems behave as the semiconductor. These results are in quite agreement with ref.^17^ in which it is reported that similar to MLAGNRs, the electronic phase of BLAGNRs depends on the ribbon width as well so that it shows metallic behavior when the ribbon width is equal to *n* = 3*p* + 2 ($p\in [1,{\mathbb{N}}]$), whilst they are semiconductor for ribbon width equal *n* = 3*p* and *n* = 3*p* + 1. However, in our modified model all phases of BLAGNRs are semiconductor. By this, for *p* = 2 and *p* = 3 as an integer number one can obtain *n* = 6 or *n* = 7 and *n* = 11 corresponding to the semiconductor AGNRs, as presented in Fig. 3 for both cases. In addition, as shown in inset panel of Fig. 3 the band gap of 7–BLAGNR is smaller than the monolayer owing to the interlayer coupling in BLAGNR as well as the quantum confinement has weaker effects at the edges of BLAGNRs for *p*_*z*_–orbitals, i.e. those form electronic clouds over and below of the layers perpendicularly^17^. Also, from Fig. 3 one can find out that both different widths of BLAGNRs illustrate two van Hove singularities around the energy ${\mathcal E} /t=\pm \,1$, while in the case of MLAGNR the single van Hove singularity is observed. On the other hand, one can clearly see the electron-hole symmetry in the states distribution, i.e. the mirror symmetry between the valence and conduction bands in all cases when there is no impurity. Further, the electronic DOS of both pristine MLAGNRs and BLAGNRs show that the number of van Hove singularities get rise with increasing the ribbon width^50^. The reason of these behaviors can be understood from this rule: Area under the DOS curve should be remained constant for electronic systems.

**Figure 4:**
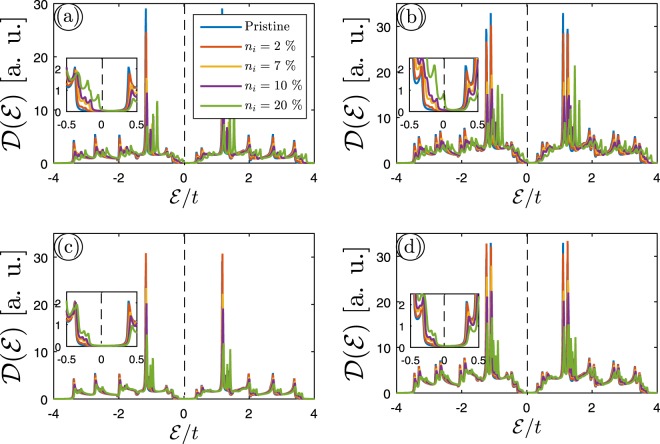
The effect of charged impurity concentration *n*_*i*_ on the electronic DOS of both sublattices equally in (**a**) 7–MLAGNR and (**b**) 7–BLAGNR. We have fixed the impurity scattering potential at *v*_*i*_/*t* = 0.5. The same effects when only one of the lattices is doped are illustrated in panels (c,d).

Now we seek to study the electronic phase of MLAGNR and BLAGNR in the presence of impurity using perturbed DOS. We first consider that the charged dilute impurity with different concentrations are added to both lattices equally under such conditions that the ribbon width and scattering potential (in units of *t*) are fixed at 7 and 0.5, respectively, as shown in panels (a) and (b). Then panels (c) and (d) demonstrate the same investigations only on one of the sublattices. The ribbon width effects will be discussed later. Figure 4 indicates the perturbed DOS of {(a), (c)} 7–MLAGNR and {(b), (d)} 7–BLAGNR as a function of *n*_*i*_. It can be seen that an increase in impurity concentration leads to a decrease in the height of the van Hove singularities, resulting in the electron-hole symmetry breaking, which in combination with tunning the band gap improve on/off ratios of the graphene devices. Moreover, the inset panels in Fig. 4 clearly show that the band gap reduces with impurity concentration because of the midgap and midband states formation for both systems. Interestingly, in a comparison of the band gap of pristine lattices with doped ones, we found that only 7–BLAGNR, i.e. panel (b) when doping both sublattices equally suffers a semiconductor-to-semimetal phase transition at strong impurity concentrations. This could be the case because of the general small band gap of BLAGNRs in comparison with MLAGNRs. However, surprisingly, a finite band gap can be observed as well at positive energy side. This is exactly the coexistence of the semimetallic and semiconducting phase at ${\mathcal E} /t=0$ and ${\mathcal E} /t > 0$, respectively. It is excellent that our results are in agreement with refs^46,51^. To compare, in ref.^46^ similar midgap states are observed in DOS of graphene with resonant (hydrogen) impurities and vacancy. They have considered a vacancy as a lattice point with infinite on-site energy, in other words, the value of its hopping parameters to other sites is zero. Also, T. O. Wehling *et al*.^51^ have investigated the effect of covalent impurities on graphene. They found that covalent impurities with one chemically active electron make midgap states that are very stable because of suppressing migration of these impurities via the electronic structure of graphene.

**Figure 5:**
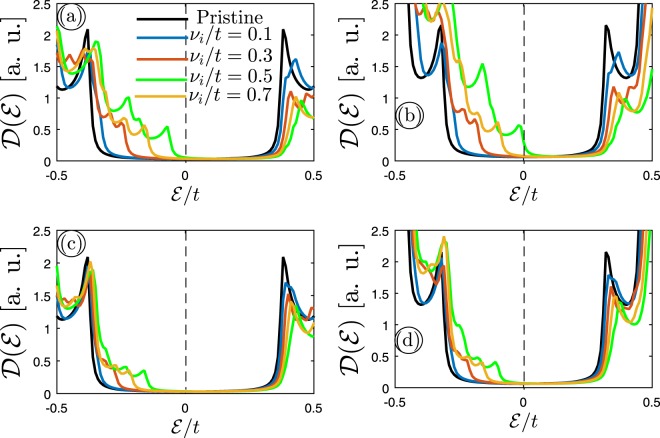
The calculated electronic DOS of (**a**) 7–MLAGNR and (**b**) 7–BLAGNR when the charged impurity scattering potential *v*_*i*_/*t* is altered for both sublattices at *n*_*i*_ = 10%. The case of doping for only one of the sublattices is shown in panels (c,d).

For the next step, we again assume that 7–MLAGNR and 7–BLAGNR are in the presence of the charged dilute impurity when doping both sublattices equally and one of them, but with this difference that the impurity scattering potential changes while its concentration is fixed at 10%. As shown before, our findings for both lattices proved that in the presence of impurity the key outcoming in perturbed DOS emerged at the low-energy limit; thus, we focus on this region to investigate the effects of impurity scattering potential changes on perturbed DOS of mentioned lattices in Fig. 5. The low-energy perturbed DOS of (a) 7–MLAGNR and (b) 7–BLAGNR as a function of ${\mathcal E} /t$ for different *v*_*i*_/*t* are shown in Fig. 5. As it is illustrated, the midgap states (very tiny peaks) become visible for both systems when *v*_*i*_/*t* is greater than or equal to 0.3, and consequently, the band gap demonstrates smaller value once this extra scattering potential is added to the carrier dynamics. This, in turn, breaks the electron-hole symmetry between the DOS curves in the left and right side of the Fermi energy. In addition, by taking a closer look at panels one can announce a phase transition from semiconductor to semimetal, albeit very weak, in 7–BLAGNR (when both sublattices are doped) when the impurity scattering potential is set to *v*_*i*_/*t* = 0.7. Therefore, charge impurity doping is a useful way to tune the band gap of *n*–MLAGNRs and –BLAGNRs as well as increase their real application in industry. Furthermore, a comparison between the panel (a) and (b) reveals that the midgap states in perturbed DOS of 7–BLAGNR are denser than ones for 7–MLAGNR due to the intensities in the vicinity of the zero energy. While in the case of (c) and (d) for doping only one of the sublattices, there is no big change between MLAGNR and BLAGNR.

Generically, in two scenarios above for the doping ways, we observed that the charged impurity made some midgap states around the Fermi level, and eventually led to the phase transition when *n*_*i*_ or *v*_*i*_/*t* was strong enough. So far, we investigated the case of MLAGNRs and BLAGNRs when the ribbon width was equal to 7. It should be noted that there is no special reason for the ribbon width choices in both cases and it can be expanded to other cases as well. In what follows, for the case of [3*p* + 2]–MLAGNRs and –BLAGNRs, we again conduct our study in two categories: we first assume that these systems are in the presence of charged impurity with the various amounts of impurity concentration and the same scattering potential. Whereas in the second scenario we have different values of *v*_*i*_/*t* for fixed *n*_*i*_. Here, we choose the ribbon width equal to 11 [*p* = 3] and carry out mentioned scenarios above for 11–MLAGNR and 11–BLAGNR in the presence of charged impurity when both sublattices and/or one of the sublattices are infected. According to the previous findings, the curves around the Fermi-level can provide main information of electronic properties of GNRs in the presence of charged impurity. Thereby, we again concentrate on the low energy region.

**Figure 6:**
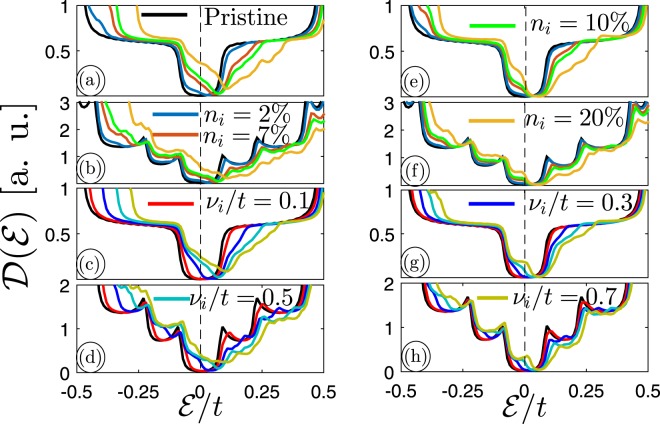
The perturbed DOS of (**a**) 11–MLAGNR and (**b**) 11–BLAGNR in the presence of different charged impurity concentration *n*_*i*_ at *v*_*i*_/*t* = 0.5. The effect of charged impurity scattering potential *v*_*i*_/*t* at fixed impurity concentration *n*_*i*_ = 10% on DOS of (**c**) 11–MLAGNR and (**d**) 11–BLAGNR. These cases are for both doped sublattices. With the same manner, the case that only one of the sublattices is infected is also investigated in panels (e–h).

The effects of charged impurity concentration *n*_*i*_ on perturbed DOS of both sublattices in 11–MLAGNR and 11–BLAGNR at fixed *v*_*i*_/*t* = 0.5 are presented in Fig. 6(a,b), respectively. Also, the low energy perturbed ${\mathscr{D}}( {\mathcal E} )$ of 11–MLAGNR and 11–BLAGNR for different impurity scattering potentials and an ascertained *n*_*i*_ equal to 10% for both doped sublattices are illustrated in panels (c) and (d), respectively. With the same manner, the effect if *n*_*i*_ and *v*_*i*_/*t* on perturbed DOS of 11-MLAGNR and 11-BLAGNR when only one of the sublattices is infected with impurity is investigated in Fig. 6(e–h). As can be seen from figures, both above systems are in the semiconductor phase with and without impurity and the mirror symmetry ${\mathscr{D}}( {\mathcal E} )={\mathscr{D}}(\,-\, {\mathcal E} )$ is broken. Moreover, we found that midband states in both energy sides appear in the presence of charged impurity originating from the electronic interaction between the host and guest electrons. This leads to a new dispersion pathway for host electrons and in turn a new proper bounding place. As explained before, the degeneracy of states at the Fermi level in BLAGNRs is generally more than the monolayer ones (See Fig. 2), which is valid also here. In addition, from panels (a)/(b) and (c)/(d) of Fig. 6 we can observe that the degenerate state at Fermi level alters by an increase in impurity concentration and scattering potential, respectively. It is worth bearing in mind that in the band structure of these lattices, the overlap of valence and conduction bands close to the Fermi level determines how the degenerate states should be formed in the electronic DOS^52^. Clearly, the semiconductor-to-semimetal electronic phase transition in the case that both sublattices are doped equally with randomly impurities is much faster than the case that only one of the sublattices is infected with impurity atoms.

**Figure 7:**
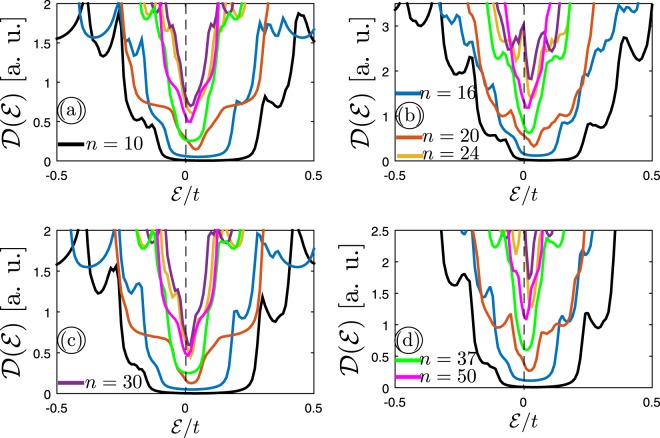
The comparison between DOS of impurity-infected semiconductor phase of (**a**) MLAGNR and (**b**) BLAGNR when the ribbon width is changed. The impurity parameters are fixed at *n*_*i*_ = 10% and *v*_*i*_/*t* = 0.5. Panels {(a,b)} and {(c,d)} refer to the case when both sublattices and one of the sublattices are doped, respectively.

As a desirable result, we report that the electronic structure of semiconductor systems is altered significantly with the impurity. For this reason, in finishing we restrict ourselves to the influence of the more realistic ribbon width of semiconductor AGNRs subjected to an impurity source with *n*_*i*_ = 10% and *v*_*i*_/*t* = 0.5 on the corresponding electronic DOS in Fig. 7. Actually, we would like to know how the ribbon width affects the phase of the semiconductor MLAGNRs and BLAGNRs. In so doing, four perturbed DOS panels (a–d) when both sublattices and one of them is infected with impurity are plotted with the same manner. It is worthwhile to mention that in our numerical calculations the integer number *p* is chosen as 3, 5, 6, 8, 10, 12, and 16 resulting in values of *n* = 10, 16, 20, 24, 30, 37, and 50, respectively. As shown in Fig. 7 we found that in the presence of impurity the values of perturbed DOS of *n*–AGNRs at zero energy become non-zero. This implies that a phase transition occurs for both lattices by increasing their width. It should be pointed out that in our formalism the ribbon width of both layers in BLAGNRs is changed simultaneously. Of course, different configurations for width of bilayer case could be chosen in the research way as well but this can be considered in our future researches. In short, we report that the sharpness of transition is more keen in impurity-infected BLAGNRs than MLAGNRs as before, while this is not the case in clean systems. For instance, in ref.^33^ it is stated that analytic scaling rules prove that in the absence of impurity the band gaps of 3*p*– and [3*p* + 1]–AGNRs are inversely proportional to the corresponding ribbon width. These findings show that an increase in width, this translates to the increase of hopping integrals between carbon atoms at the edges, leads to decrease of band gap. The existence of band gap means that the system is still in the semiconducting phase, whilst we have a semiconductor-to-semimetal phase transition when the impurity is doped randomly to the systems.

**Figure 8:**
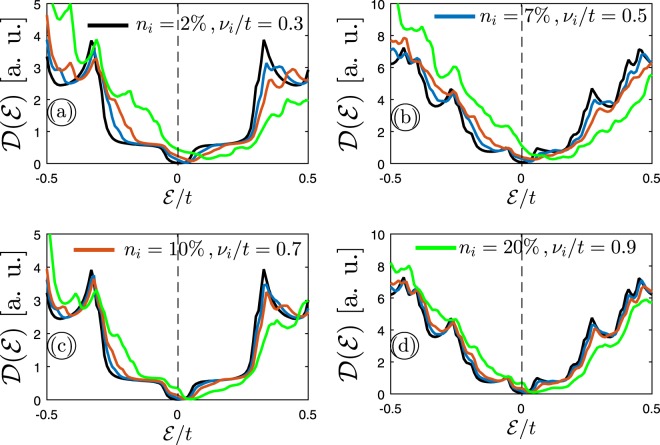
The calculated electronic DOS of (**a**) 11–MLAGNR and (**b**) 11–BLAGNR when the charged impurity scattering potential *v*_*i*_/*t* and impurity concentration *n*_*i*_ are irrelevant on both sublattices. The same study for one of the sublattices is presented in panels (c,d).

In the last paragraph of this section, we present the results of the third doping way, i.e. the case when both *n*_*i*_ and *v*_*i*_/*t* are irrelevant. Figure 8(a,b) show the perturbed DOS of 11–MLAGNR and 11–BLAGNR when both sublattices are doped with irrelevant impurity concentration and scattering potentials. As before, Fig. 8(c,d) mention the results of perturbed DOS in 11–MLAGNR and 11–BLAGNR, respectively, when one of the sublattices is doped with irrelevant impurity characters. Compared to the two previous doping ways, the electron-hole symmetry is much more sensitive and the changes are more evident in this way, which is expectable because the configuration of propagating electronic waves when both impurity characters are irrelevant is much more than the cases when only one of them affects the spatial distribution of electronic waves.

## Conclusions

To sum up, we have numerically studied the effects of charged dilute impurity on the electronic structure of mono- and bi-layer GNRs with armchair shaped edges, aimed at increasing their real applications through tuning of the band gap. To this end, we have calculated the impurity-infected DOS of mentioned systems using the modified tight-binding Hamiltonian model in the presence of edge effects, the Born approximation, and the Green’s function method. In so doing, we consider the MLAGNRs and BLAGNRs with two different ribbon widths for the semiconducting phases. The findings of our study showed that in the presence of charged dilute impurity some *midgap* states became visible in the perturbed DOS of semiconducting type of both lattices. And eventually, a phase transition from semiconductor to semimetal emerged at strong impurity concentrations and/or impurity normalized scattering potentials for both MLAGNRs and BLAGNRs. This, in turn, leads to a remarkable point: Coexistence of semiconducting and semimetallic phases in the system. Further, because of the induced-impurity states, the electron-hole symmetry breaks down in both semiconducting AGNRs. Finally, we have reported that by increasing the value of the ribbon width, the impurity-infected DOS of the semiconducting version of AGNRs illustrates a semiconductor-to-semimetal phase transition for both mono- and bi-layer systems, which this is not the case in the pristine system.
